# Genomic identification of conservation areas amid lineage divergence and admixture in a threatened island gecko

**DOI:** 10.1186/s12915-025-02394-6

**Published:** 2025-10-22

**Authors:** Richard P. Brown, Luca Bianco, Paolo Fontana, Peter Shum, Raquel Vasconcelos, Yuanting Jin

**Affiliations:** 1 School of Biological & Environmental Sciences, Liverpool John Moores University, Liverpool, L3 3AF UK; 2 Research and Innovation Centre, Fondazione Edmund Mach, 38098 San Michele all′Adige, Italy; 3 BIOPOLIS Program in Genomics, Biodiversity and Land Planning, InBIO Laboratório Associado, Universidade Do Porto, 4485-661 Vairão, Portugal; 4 Department of Life Sciences, University of Coimbra, 3004-504 Coimbra, Portugal; 5 College of Life Sciences China, China , Jiliang University, Hangzhou, 310018 China

**Keywords:** Admixture, Conservation, Dating, Divergence, Evolution, Genome, Introgression, Island, Lizard, Phylogenomics

## Abstract

**Background:**

Identification of ancient evolutionary lineages and areas of natural admixture can have important implications for conservation policies aimed at preserving biodiversity in the face of existential threats. The island gecko *Tarentola boettgeri* is potentially threatened by the introduced California kingsnake (*Lampropeltis californiae*) within the relatively small oceanic island (1532 km^2^) of Gran Canaria, Canary Islands, Spain.

**Results:**

A 1.9-Gb *T. boettgeri* genome was assembled de novo from PacBio HiFi reads. Sequences obtained by genotyping-by-sequencing (GBS) were aligned to this reference and provided over 440,000 SNPs from 134 geckos obtained from 40 sample sites (Fig. 1). Analyses of ancestry coefficients supported five genomic groups within Gran Canaria plus two additional groups from the other parts of its range, namely the Canary Island of El Hierro and the Selvagens archipelago. Phylogenomic and divergence time analyses of both GBS sequences and SNPs revealed lineage divergence within Gran Canaria, starting 1.5–2.9 Ma, and also between-island divergence due to subsequent colonization of both the Selvagens and El Hierro around 1 Ma. The latter two colonization events occurred from distinct lineages that had originated in the NW and the W of Gran Canaria, respectively. Lineage divergence within Gran Canaria appears to have been followed by secondary contact and admixture, likely starting in the Late Pleistocene around 20–110 Ka ago. Individuals with significant mixed ancestry appear to be limited to as little as 5 km either side of contact zones. This facilitates identification of sites containing individuals with negligible mixed ancestry for each of the five ancient lineages.

**Conclusions:**

The ability to genomically identify five ancient Gran Canarian lineages and geographical areas with ostensibly low mixed ancestry provides a foundation for practical conservation actions—such as selecting sites for creation of snake exclusion areas and/or the acquisition of individuals for ex situ captive breeding. These actions will help conserve the extensive within-island diversity in this species.

**Supplementary Information:**

The online version contains supplementary material available at 10.1186/s12915-025-02394-6.

## Background

Genomics significantly contributes to conservation by enabling the delimitation of biodiversity, often through the identification of new lineages [1, 2]. While this can be achieved using genetic analyses of a small number of loci, genome-wide data offer greater reliability by providing numerous locus histories which help avoid errors due to effects such as incomplete lineage sorting [3–5].

Another potentially important application of conservation genomics is the identification of admixture where divergent lineages meet at regions of secondary contact. Here, hybridization and backcrossing lead to hybrid zones containing individuals of mixed ancestry [6]. These zones can be identified by analyses of genomic data [7]. The conservation importance of these studies may be quite obvious in cases where contact between lineages has been mediated anthropogenically, such as between native and invasive species [8]. However, naturally occurring hybrid zones should also be incorporated within conservation strategies, particularly those designed to preserve the genomic integrity of the parental lineages. For example, where hybrid individuals have lower fitness, it is beneficial to create protected areas at locations where there is little or no admixture. The primary aim here was to examine whether areas of low admixture could be identified for multiple lineages within a restricted geographical space.

The study organism, Boettger’s wall gecko (*Tarentola boettgeri*), exhibits ancient divergence within a small island and is of conservation concern due to the existential threat that may be posed by a recently introduced snake predator. The gecko is currently found from sea level to elevations over 1550 m on Gran Canaria (1532 km^2^, elevation 1956 m, 27.9714 N, 15.5904 W), Canary Islands, and is locally abundant, with no obvious major gaps in its distribution (RPB personal observation). A previous study of *T. boettgeri* reported five ancient within-island mtDNA lineages of Miocene origin [9]. As in several other examples of cladogenesis within volcanic islands [10–12], lineage divergence appears to have been mediated by populations becoming isolated within refugia during Pliocene/Miocene volcanic events, such as major volcanic eruptions and debris avalanches [9, 13]. Secondary contact between the divergent lineages, following range expansion out of isolated refugia, is facilitated by the limited island area [14, 15]. The spatial structuring of mtDNA lineages appears to be parapatric, suggesting limited admixture following secondary contact [9, 16].

The California kingsnake, *Lampropeltis californiae*, was first recorded on Gran Canaria around 1998, and its rapid range expansion has been linked with population reductions in all three native lizards [17–19]. Populations of the endemic lacertid, *Gallotia stehlini*, and endemic skink, *Chalcides sexlineatus*, appear to be particularly affected [18], and their IUCN assessments were recently updated to Critically Endangered and Endangered, respectively [20, 21]. *T. boettgeri* presently appears less impacted by *Lampropeltis* [18], and its IUCN listing remains Least Concern [22]. However, the lower assessment category is also likely due to *T. boettgeri* being the only one of the three Gran Canarian lizards that naturally occurs elsewhere: it is also native to the island of El Hierro (Canary Islands, Spain), 230 km to the west, and the uninhabited Selvagens archipelago (Portugal), or “Savage Islands”, approximately 160 km to the north [23, 24]. The latter two populations have been described as distinct subspecies (*T. b. hierrensis* and *T. b. bischoffi*, respectively [25, 26]) to reflect divergence from Gran Canarian populations. Nonetheless, mtDNA analyses suggest that these islands were colonized *after* within-island divergence in Gran Canaria [9, 23, 24], indicating that this subspecific taxonomy does not adequately capture the diversity within *T. boettgeri*.

The current conservation strategy by the local governmental environmental protection agency (Cabildo Insular de Gran Canaria) includes provision of natural *Lampropeltis* exclusion areas to protect the three native lizards. We argue that all ancient lineages should be preserved, and that the number and locations of exclusion areas should be strategically designed to support this goal. This should extend across all threatened species. For example, *Chalcides sexlineatus* shows very clear intraspecific morphological [27, 28]), mtDNA [12, 29], and nuclear microsatellite [12] structuring within the same island, with molecular divergence originating from the Pleistocene, which supports establishment of two or more geographically distinct exclusion areas. The optimal strategy for *T. boettgeri* similarly depends on the number of within-island lineages, their antiquity, and levels of admixture between them, which will be examined here.

**Fig. 1:**
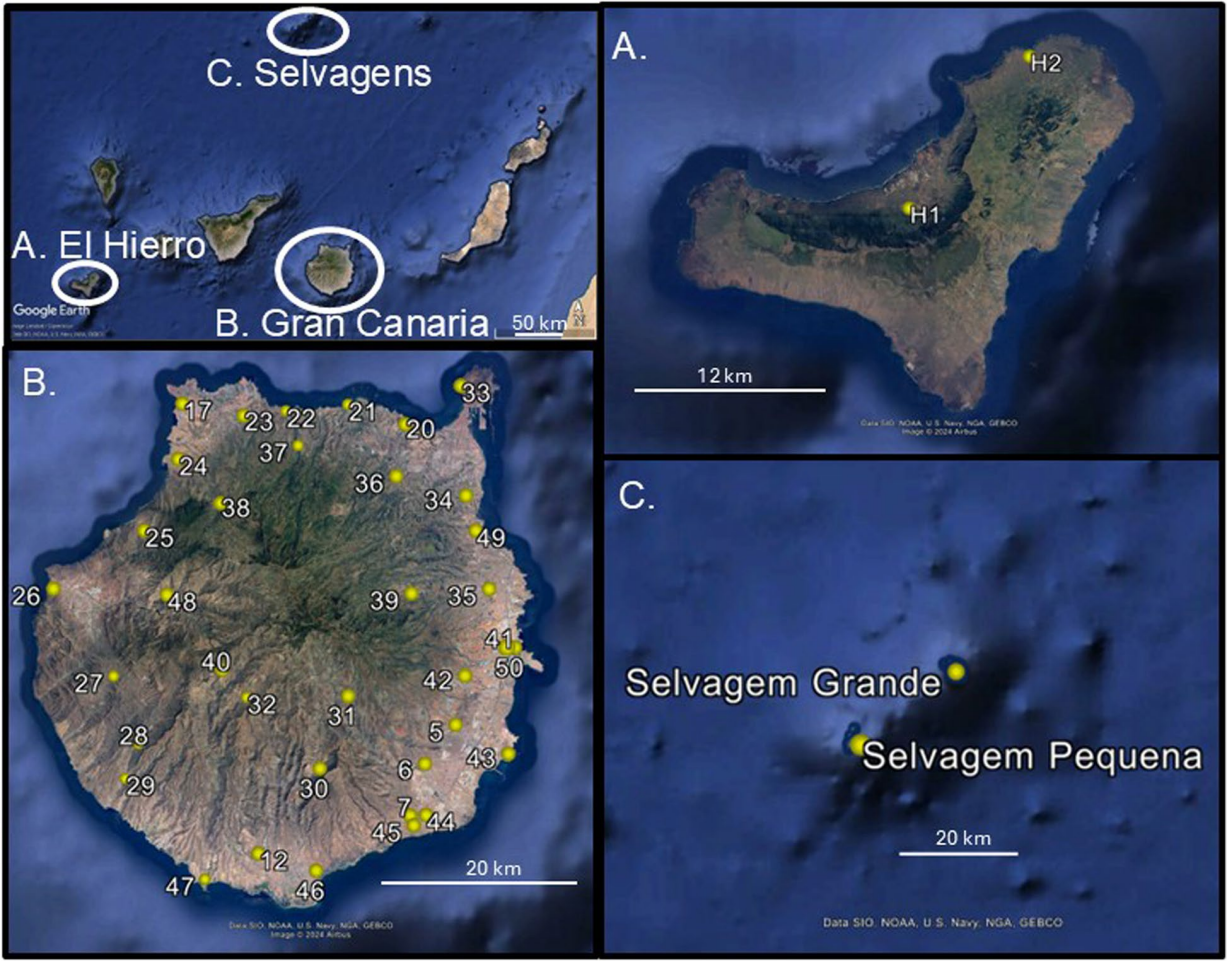
Study area (top left) and magnified images of each island (group) with locations of all sample sites. Additional file: Fig. 1 contains details on latitudes, longitudes and sample sizes. Map data: Google, SIO, NOAA, U.S. Navy, NGA, GEBCO

We used the nuclear genome to infer lineage divergence and examine the hypothesis of limited admixture among Gran Canarian populations. The rationale is that ancient lineage divergence (as suggested by the mtDNA [9]) will have led to increased genetic incompatibilities [30, 31]. Low levels of admixture could mean the existence of single-ancestry populations, despite the very limited island area. Our second aim was to describe the biogeographical history of this species, in terms of within-island divergence and between-island colonization (Fig. 1).

## Results

### De novo whole genome sequencing

The genome size was estimated as 1.9 Gb (Additional file: Fig. S1). The assembly process produced two sets of highly contiguous sequences (one for each haplotype): haplotype 1 contained 700 contigs (maximum size 57.1 Mb, total size: 2.088 Gb, N50: 8.7 Mb), while haplotype 2 contained 548 contigs (maximum size: 11.7 Mb, total size: 2.116 Gb, N50: 11.8 Mb) [32]. The BUSCO analysis indicated (i) high levels of completeness of the genic spaces of each haplotype (93.6% for haplotype 1 and 94.6% for haplotype 2), (ii) low levels of fragmentation (1.1% fragmentation for haplotype 1 and 1.2% fragmentation for haplotype 2), and (iii) a small number of missing groups (5.3% missing for haplotype 1 and 4.2% missing for haplotype 2).

A total of 99.37% of the k-mers present in the reads were also present in either haplotype 1, haplotype 2, or both (Additional file: Fig. S2), according to the merqury analysis.

For haplotypes 1 and 2, a total of 38 and 41 contigs, respectively, contained telomeric sequence at one end. We identified 30,650 coding genes in haplotype 1 and 30,822 in haplotype 2. The BUSCO analysis of gene prediction showed a poorer completeness of the gene space with 88.3% (6.6% fragmented) genes identified for haplotype 1 and 88.5% (6.3% fragmented) identified for haplotype 2.

### Alignment of GBS reads

Haplotype 2 of the de novo genome was used as the reference genome for alignment of GBS reads [32]. SNP calling with *ipyrad* initially provided 526,082 SNPs, although this was reduced to 440,336 SNPs after filtering with VCFtools. The thinned dataset (one SNP per locus) derived from the latter set of SNPs contained 18,158 SNPs.

### Analyses of introgression

**Fig. 2:**
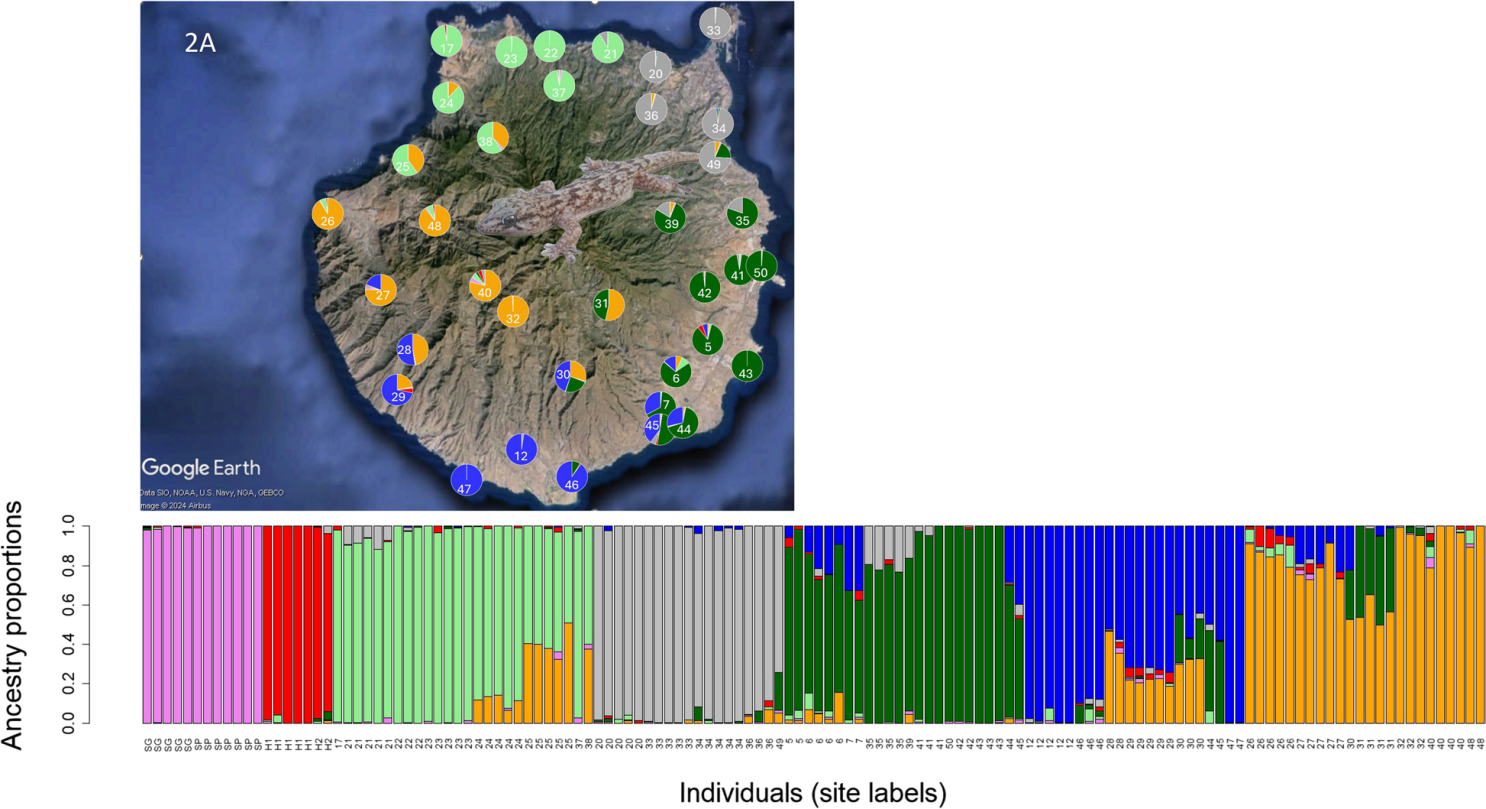
LEA admixture analysis for *K* = 7 genomic clusters (bottom figure), showing ancestry proportions of every sampled individual. The top diagram (2A) shows overall ancestry proportions at each of the Gran Canaria sample sites. SG is Selvagem Grande, SP is Selvagem Pequena, and H1 and H2 are the two sample sites on El Hierro. All other sites (labelled by number only) are on Gran Canaria. Map data: Google, SIO, NOAA, US Navy, NGA, GEBCO

Mean cross-entropies obtained from LEA replicates were lowest for *K* = 5–7 clusters (Additional file: Fig. S3). For subsequent analyses, we assumed *K* = 7 ancestral populations because of the following: (i) the single lowest cross-entropy value across all replicates was observed for *K* = 7, (ii) previous descriptions of mtDNA groups with similar distributions indicated seven major groups within *T. boettgeri*, and (iii) the Selvagens, El Hierro, and Gran Canaria individuals were separated into distinct clusters when *K* ≥ 6. Ancestry coefficients are shown graphically in Fig. 2.

BPP analyses of introgression among the areas occupied by the five Gran Canarian genomic clusters supported the mixed ancestry estimates obtained by the LEA analysis (Table 1). Sites that were spatially intermediate between sites with individuals of differing single ancestries contained individuals with mixed ancestries. These mixed ancestry individuals mostly showed relatively similar introgression probabilities from each of the two single ancestry sites. Nonetheless, there were some exceptions. Individuals from site 21 on the north coast — half-way between single ancestry sites 20 (NE group) and 22 (NW group) — showed an introgression probability from the NW of 81%. But overall, single ancestry populations that were separated by distances of 11 to 31 km were generally found to provide similar genetic contributions to intermediately located populations. None of the 95% highest posterior densities (HPD) for the time *τ*_I_, representing the hybridization time of the two specified groups in each comparison, included zero (Table 1). Using subsequently estimated mean rates of molecular evolution (see “Divergence times”), the mean estimates for the times of contact/hybridization corresponded to values ranging from 20 Ka ago for contact of the NE and E groups to 118 Ka ago for the W and NW groups.

**Table 1:** Introgression probabilities (φ) and their 95% HPDs for selected samples (I) that were intermediately located between two sites with single ancestry that correspond to two different genomic groups (denoted in first two columns) within Gran Canaria. For example, φ_A→I_ for the analysis of sites 22 (corresponding to the NW genomic group), 20 (corresponding to the NE genomic group) and 21 (intermediate site) indicates an introgression probability of 0.806 [95% *HPD*: 0.782, 0.829] into site 21 from site 22. Mean posterior hybridization time (and 95% HPD) is *τ*_I_ (measured in expected number of mutations per site). The introgression probabilities of group B into I (*φ*_B→I_) are equal to (1–φ_A→I_) but provided for completeness

Group A (site)	Group B (site)	Intermediate site(s): I	Distance: A-I (km)	Distance: B-I (km)	τ_I_	φ_A→I_	φ_B→I_
NW (22)	NE (20)	21	5.7	5.7	4.9 × 10^−5^[2.3 × 10^−5^, 7.4 × 10^−5^]	0.806 [0.782, 0.829]	0.194 [0.171, 0.218]
NE (34)	E (41)	35	8.8	5.4	1.0 × 10^−5^ [4.0 × 10^−6^, 1.6 × 10^−5^]	0.342 [0.292, 0.392]	0.658 [0.608, 0.708]
E (43)	S (12)	7, 44, 45	10.2	14.7	1.1 × 10^−5^ [5.0 × 10^−6^, 2.1 × 10^−5^]	0.542 [0.489, 0.593]	0.458 [0.407, 0.511]
E (42)	W (32)	31	10.7	9.3	5.5 × 10^−5^ [5.0 × 10^−6^, 1.2 × 10^−4^]	0.545 [0.485, 0.601]	0.455 [0.399, 0.515]
S (12)	W (32)	29	14.0	13.4	1.6 × 10^−5^ [3.0 × 10^−6^, 3.7 × 10^−4^]	0.779 [0.739, 0.817]	0.221 [0.183, 0.261]
W (32)	NW (23)	25	17.8	14.0	5.8 × 10^−5^ [1.4 × 10^−5^, 9.8 × 10^−5^]	0.379 [0.289, 0.472]	0.621 [0.528, 0.711]

### Evolutionary history

**Fig. 3:**
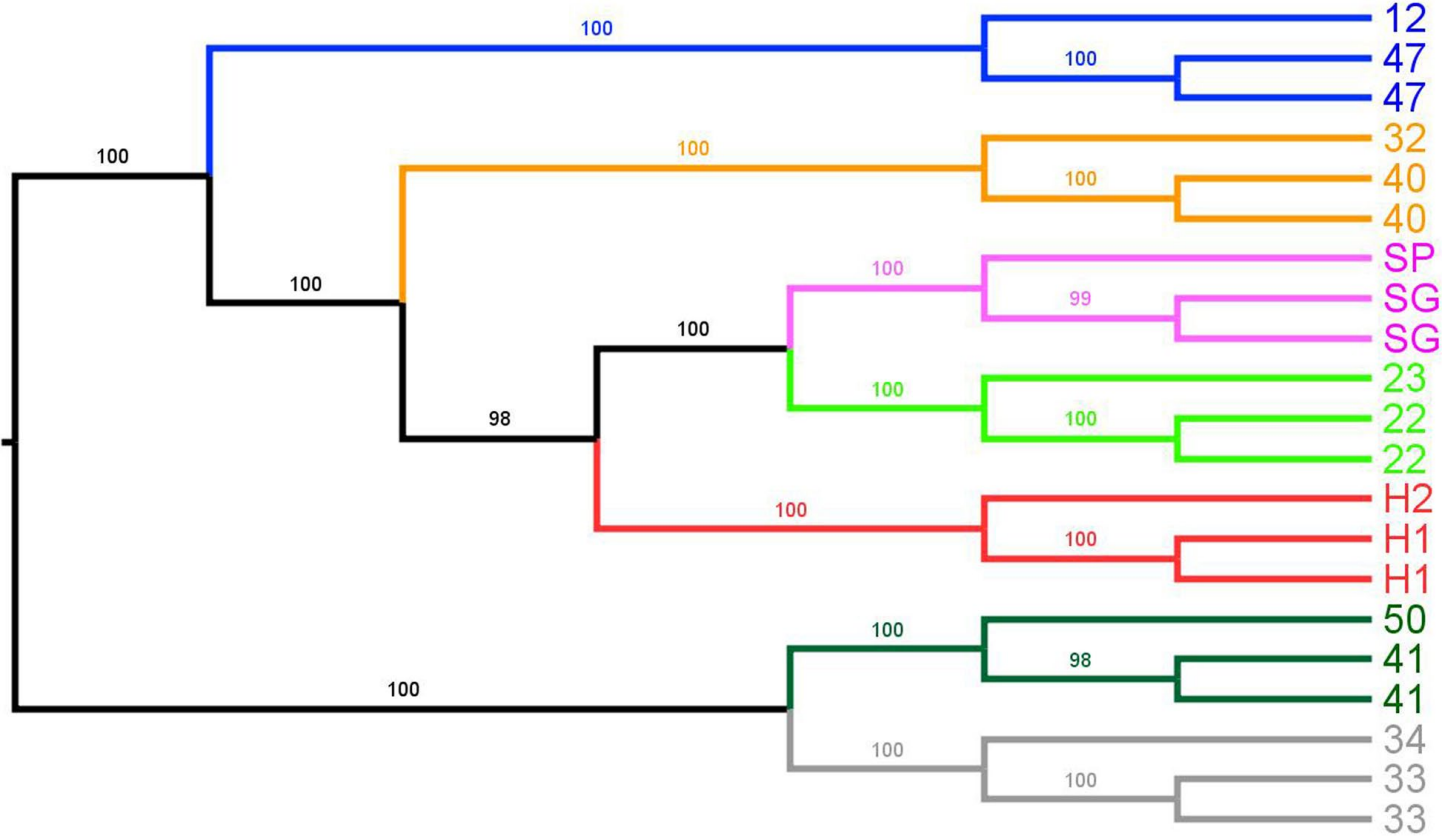
Maximum likelihood tree of individuals with bootstrap values on internal nodes. Tips represent the sample site labels provided in Fig. 1 and Additional file: Table 1

The seven genetic clusters identified by the LEA analysis were well-supported (bootstrap support > 98%) as seven major lineages in the ML tree from SNP genotypes (Fig. 3). Two groups originated from the most basal split within *T. boettgeri*. One of these contained NW, W, and S Gran Canarian and Selvagens and El Hierro individuals, while the other contained E and NE Gran Canaria individuals. The former indicated a sister-group relationship between the Selvagens and the NW Gran Canaria group. These two groups were sister lineages to the El Hierro group, which was in turn outgrouped by the W Gran Canaria group and then by the S Gran Canaria group.

**Fig. 4:**
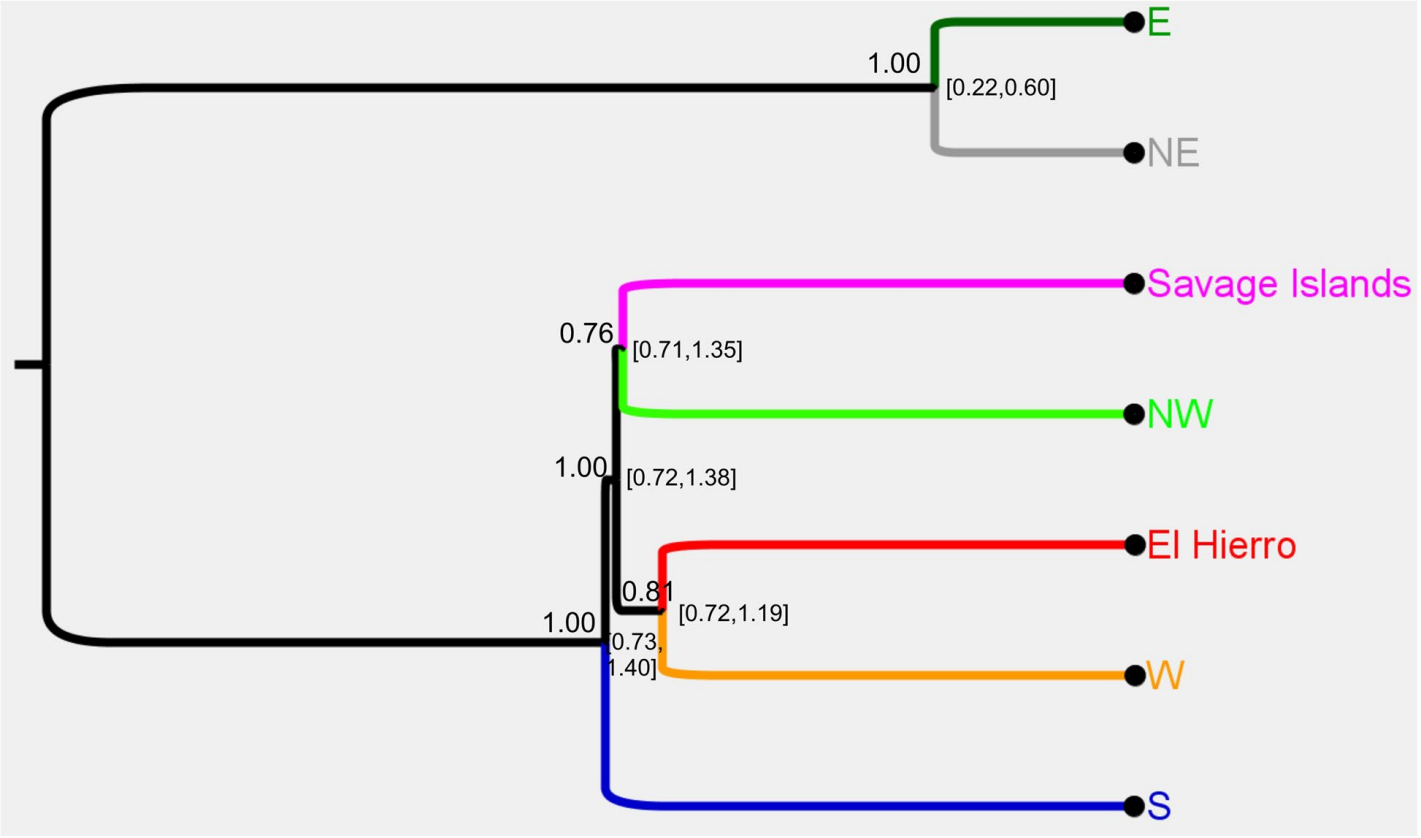
BPP species tree for all genomic groups within *T. boettgeri*. Values to the upper left of nodes represent posterior clade credibility values, while values to the right of the nodes in parentheses represent posterior intervals on divergence times (Ma)

Posterior BPP species trees all supported the same two major evolutionary lineages detected by the SNP analysis of individual genotypes, i.e. one group from NW, W, and S Gran Canaria, Selvagens, and El Hierro and the other from E and NE Gran Canaria, with the S Gran Canaria group always showing the earliest split within the former (Fig. 4). There were three topologies with a posterior probability (PP) > 0.05, and these differed only in terms of relationships within the lineage containing NW and W Gran Canaria, Selvagens, and El Hierro populations. By far, the most strongly supported topology (T1; *PP* = 0.72) grouped the Selvagens with NW Gran Canaria and El Hierro with W Gran Canaria, i.e. the overall topology was as follows: ((E Gran Canaria, NE Gran Canaria), (S Gran Canaria, ((El Hierro, W Gran Canaria), (Selvagens, NW Gran Canaria)))). Support for the topology with the second highest PP was relatively weak (T2; *PP* = 0.010) and provided the following relationships within the most diverse lineage: (NW Gran Canaria, (W Gran Canaria, (Selvagens, El Hierro))). Similarly, the topology with the third highest PP (T3; *PP* = 0.07) provided the following relationships: (NW Gran Canaria, (Selvagens, (W Gran Canaria, El Hierro))). Topologies T1–T3 all showed slight differences to the ML topology computed on the concatenated SNPs.

The stepping-stone calculations used to obtain marginal likelihoods of species tree models strongly favoured the most highly supported tree, T1, over the alternative trees T2 and T3. The Bayes factor for T2 over T1 was < 0.0001 (the relative PPs of T1 and T2 were 1.0000 and < 0.0001, respectively). The Bayes factor for T3 over T1 provided the same result (to four decimal places).

The SVDquartets species tree analysis using SNPs also supported the T1 species tree topology. Bootstrap support values were 100% on all internal nodes of this tree.

### Divergence times

The oldest mean posterior divergence time was 2.20 Ma with a 95% HPD of 1.53–2.87 Ma (Fig. 4). This represented the oldest split and occurred between the group containing the E/NE Gran Canarian groups and the group containing all other lineages. The most recent split among the seven groups is estimated to be that between the E and NE Gran Canaria groups, dated at 0.40 Ma (95% *HPD*: 0.22–0.60 Ma). The estimated divergence time between the Selvagens and NW Gran Canaria populations is 1.03 Ma (95% *HPD*: 0.71–1.35 Ma), which overlaps the posterior for divergence of the El Hierro group from Western Gran Canaria (calibrated node: 0.70–1.20 Ma). The 95% HPD for the global substitution rate in this analysis was as follows: 3.58 × 10^−4^–6.70 × 10^−4^ substitutions/site/Ma.

## Discussion

**Fig. 5:**
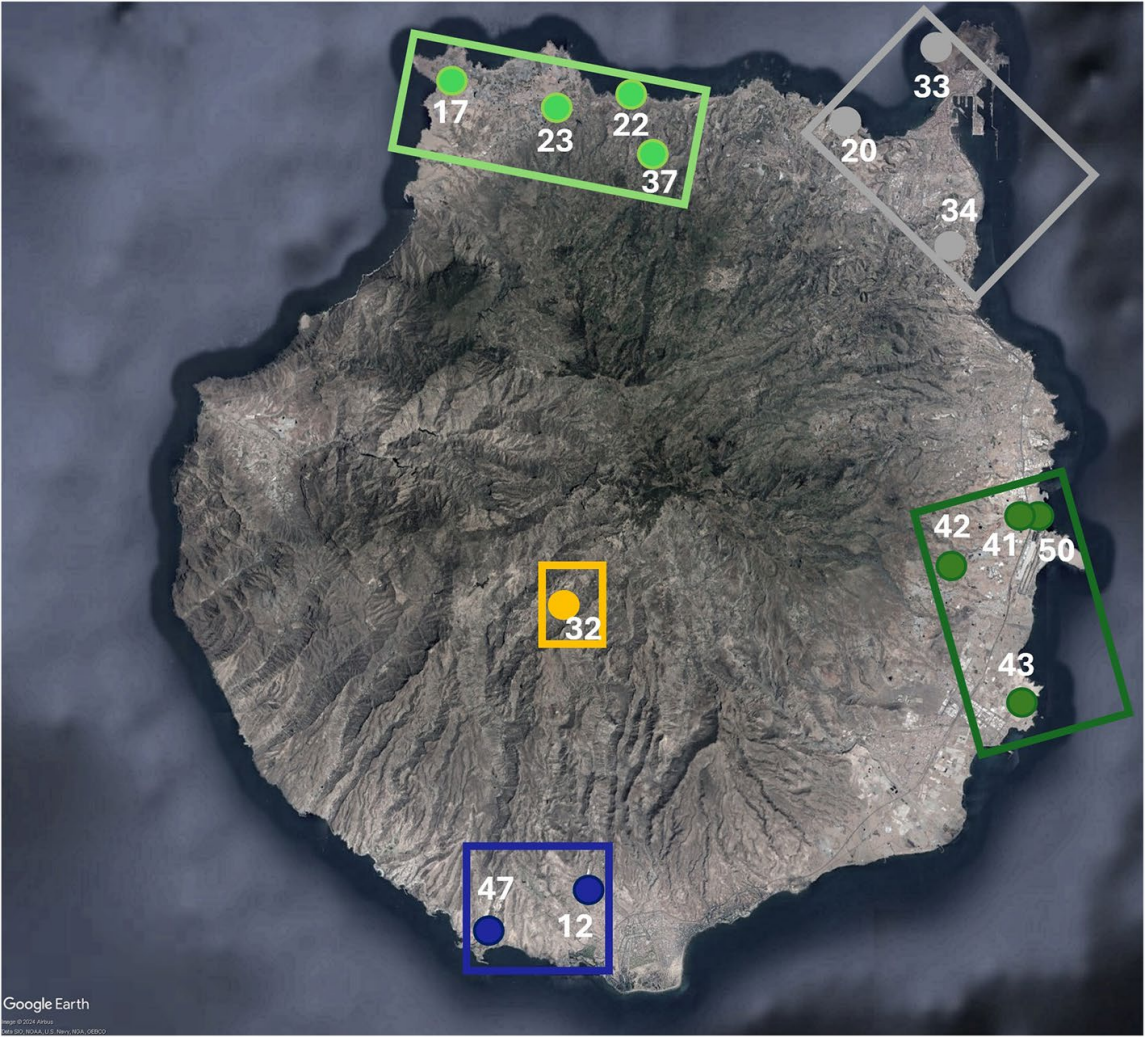
The sample sites at which sampled individuals showed ≥ 95% ancestry for a single genomic cluster, on average, suggesting negligible introgression which might make them more suitable for conservation programmes. Map data: Google, SIO, NOAA, US Navy, NGA, GEBCO

Gran Canaria has a diameter of approximately 50 km. Within this small island, there are five parapatric lineages of *T. boettgeri* that likely originated and subsequently underwent secondary contact in the Pleistocene. Mixed ancestry was detected at all areas of secondary contact where lineages meet, but single ancestry was generally detected at sample sites further away (generally 5–15 km) from these hybrid zones. Given the threat posed by the introduced California kingsnake, we argue that conservation of representative non-admixed populations is important because it helps conserve the integrity of the five ancient lineages and mitigates the potential problem of lower hybrid fitness (discussed below). Hence, the proposed conservation strategy to construct snake exclusion zones for protection of the three native Gran Canarian lizards (Cabildo Insular de Gran Canaria, pers. comm.) should primarily focus on the five areas with negligible admixture identified here (Fig. 5) in order to help preserve intraspecific diversity.

As stated previously, each of the three native Gran Canarian squamates has a different IUCN status, with Least Concern assigned to *T. boettgeri*. Nonetheless, there are at least two reasons why this should be reviewed. First, if *Lampropeltis* predation substantially lowers *Gallotia stehlini* and *C. sexlineatus* densities, then prey switching could potentially increase the threat for *Tarentola*. Second, the IUCN Red List is intended to inform biodiversity conservation using largely species-level taxonomies and does not capture the major intraspecific diversity described here. IUCN recognition of the described lineages would improve this. We also note that not all of the Gran Canaria lineages are worthy of the same conservation priority. For example, the western lineage is currently less exposed to *Lampropeltis* than the other lineages, although this may change with ongoing *Lampropeltis* range expansion. As a side note, we also draw attention to the major morphological [27, 28] and genetic [12, 29] structuring that also exists within the Gran Canarian skink (*C. sexlineatus*), favouring IUCN recognition of intraspecific diversity in this species too.

A key finding with respect to Our proposal of the five individual lineages was that levels of introgression appear relatively low. Divergent populations, each ostensibly consisting of single ancestry, are separated by only 10.4 km in one case. The within-island lineages diverged in the last 0.2–2.7 Ma, with secondary contact also likely to have commenced in the Late Pleistocene, so it is reasonable to assume that their geographical structuring has remained relatively stable. Even under very low dispersal rates without selection against hybrids, the hybrid zone would be expected to continually widen over time, leading to the creation of a single homogeneous genomic group [33]. Well-studied hybrid zones that appear to be maintained by selection against hybrids, such as those in *Bombina* toads, are of a similar width to those here [34]. The likely age and narrowness of the *Tarentola* hybrid zones therefore appear to be consistent with lower hybrid fitness, further supporting our argument that exclusion zones should be sited in areas of no admixture.

It should be borne in mind that the LEA algorithm aims to divide genotypes into distinct clusters, and so it will generally assign single ancestry to one or more individuals in each cluster and potentially underestimate mixed ancestry. Nevertheless, we show that it is an extremely useful tool for conservation genomics when the primary goal is the identification of the least-admixed populations. It is also interesting that there was no obvious relationship between the degree of genetic divergence between lineages and the degree of introgression, except perhaps for slightly lower introgression between the NE and NW lineages (which originated from the earliest within-island lineage divergence). Again, this could be investigated with more extensive sampling in the future.

**Fig. 6:**
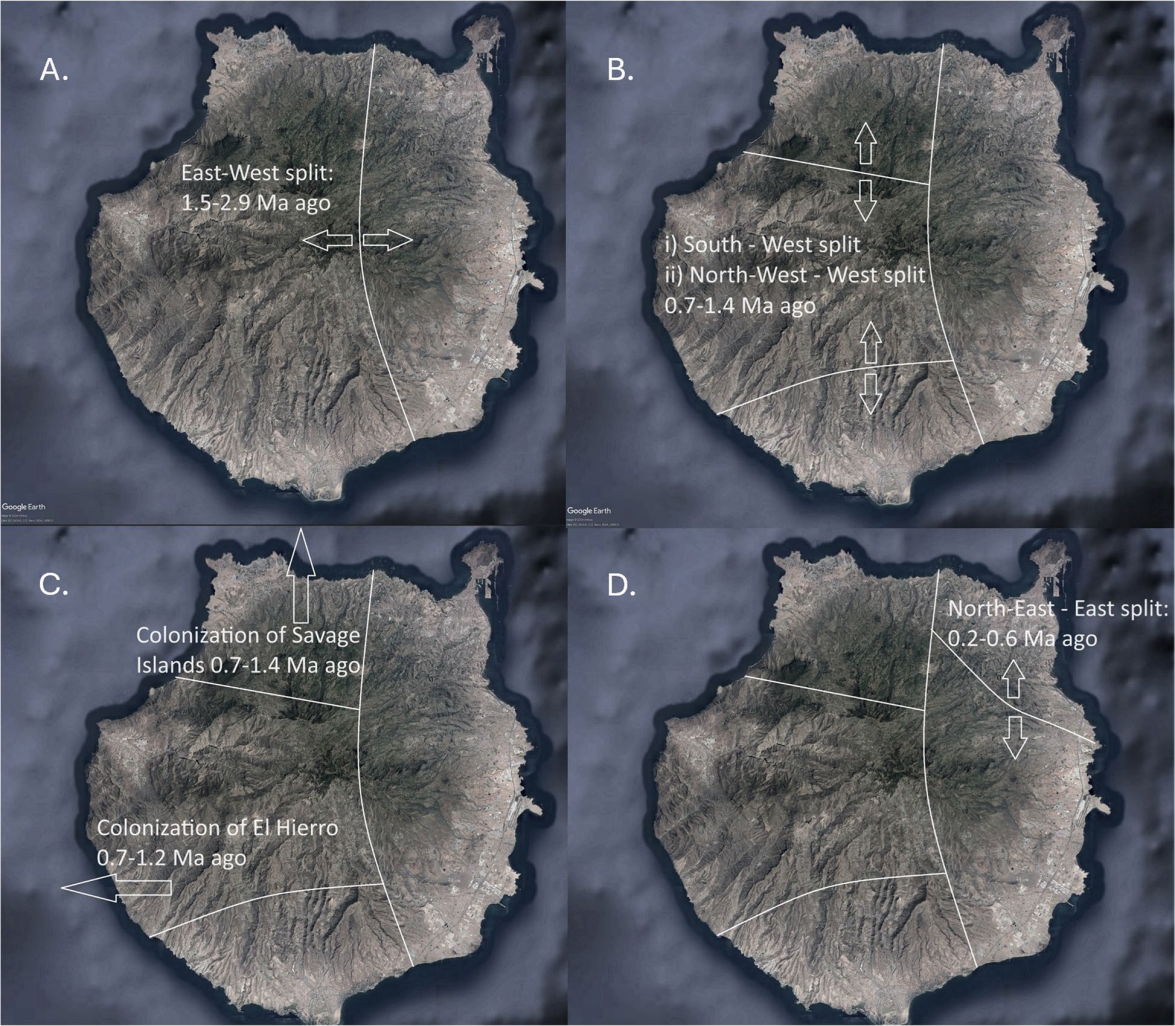
Graphical summary of the main *T. boettgeri* splitting events inferred from Bayesian divergence time dating using genomic sequences. **A** Early east–west split. **B** Two subsequent splits at a similar time within the western lineage. **C** Colonization of the Selvagens and El Hierro around the same time as events shown in subfigure **B**. **D**. The most recent split, within the eastern Gran Canarian lineage. Map data: Google, SIO, NOAA, US Navy, NGA, and GEBCO

*T. boettgeri* shows an interesting biogeographical pattern (historical events are summarized in Fig. 6). Evidence of a Late Pliocene/Early Pleistocene split was detected between eastern and western populations within Gran Canaria. This coincided with a period of eruptive activity confined to the north and east of the island 3.1–1.7 Ma [35]. Similar to *C. sexlineatus* on the same island [12], this activity may have led to isolation and divergence of eastern and western populations. Five subsequent splits within the western lineage occurred around the mid-Pleistocene and involved the following: (i) north–south within-island divergence (leading to three major Gran Canarian lineages) and (ii) colonization of the island of El Hierro and the Selvagens archipelago. The timing of these splits coincided with mid-Pleistocene volcanism within Gran Canaria, although in this case the location of the volcanic activity does not show an obvious correspondence with the spatial distributions of the lineages. El Hierro also appeared at this time and was subsequently colonized from Gran Canaria. The synchronous colonization of the Selvagens was possible given that subaerial islands are likely to have been continuously present within this archipelago since at least 4 Ma ago [36]. The most recent within-island split, between northern and eastern lineages, followed the main appearance and formation of La Isleta in the north-east of the island, some 700 Ka [37]. La Isleta originally appeared as a small independent island but was subsequently connected to Gran Canaria by sedimentation [37]. It is possible that colonization of this islet followed by expansion back into the main island played a role in the north-east split.

How do these findings differ from those inferred from mtDNA? The geographical distributions of the five previously identified Gran Canaria mtDNA lineages are quite similar to the nDNA lineages described here [9]. However, mtDNA does not allow detection of mixed ancestry. Also, the previous study could not effectively assess mtDNA introgression between different populations due to a different sampling design. A more recent intensive study of E and S lineages suggested that mtDNA introgression between populations is likely to be lower than nDNA introgression found here [16]. For example, only one (E or S) mtDNA lineage was detected at each site for individuals sampled from two sites (*n*_E_ = 15 and *n*_S_ = 30, respectively) only 3.3 km apart [16]. A narrower hybrid zone for mtDNA compared with nuclear markers has also been described for other species such as small *Bombina* toads [38].

Previous mtDNA studies have not included all *T. boettgeri* lineages, which slightly hampers direct comparisons with current findings. However, the historical relationships we detect appear to be broadly similar to previously described mtDNA relationships but with some biogeographically significant differences. The differences are confined to the major western lineage containing the Selvagens/NW Gran Canaria/El Hierro/W Gran Canaria sublineages. The Selvagens and El Hierro populations were identified as sister-mtDNA lineages [23, 24, 39] and also reported to be outgrouped by the W Gran Canaria lineage [23, 24, 39]. However, these studies did not include the NW Gran Canaria mtDNA lineage which is closely associated with the Selvagens lineage [9]. Our most strongly supported topology displays this NW/Selvagens sister-lineage relationship but also indicates that El Hierro and W Gran Canaria are sister lineages. This topology appears quite well-supported and is biogeographically more intuitive than previous findings because it does not imply that El Hierro was colonized from the Selvagens. However, it may be premature to completely rule out alternative tree topologies. Our divergence time dating suggests that the three major divergence events within the western lineage occurred over a relatively short period (see below) which means that an irrefutable consensus on the chronological order of lineage divergence events from genetic/genomic data may not be achievable.

Our estimated divergence times are more recent than those estimated from mtDNA. For example, the most basal node, corresponding to a split within Gran Canaria, has been estimated at 4.6–11.0 Ma [9] and 6.4 Ma [24], predating the 1.5–2.9 Ma 95% posterior density here. Consistency of substitution rates across related taxa gives considerable credence to dating estimates obtained from the mtDNA locus, particularly when divergence times estimated using established molecular rates coincide with major geological events that potentially mediated the divergence [40, 41]. Why should 2000 nuclear loci provide such different divergence estimates to mtDNA? Ancient introgression and recombination could potentially reduce observed nuclear DNA divergence, unlike mtDNA divergence, and therefore explain the difference. However, this would lead to a low global substitution rate. It would also be expected to cause greater relative observed divergence in El Hierro and Selvagens populations, with respect to Gran Canarian lineages (leading to a major change in tree topology), given that these diverged in isolation without admixture. Neither of these corollaries is strongly supported. For example, the nuclear rate estimated using the El Hierro calibration (posterior mean 4.9 × 10^−4^ subs/site/Ma) is quite typical for Squamata and exhibits a ratio to the *Tarentola* mtDNA rate found by previous studies that is quite similar to nDNA-mtDNA rate ratios in other taxa [42]. Instead, methodological differences almost certainly had some impact. Previous mtDNA studies have estimated sequence divergence times using substitution rates and so do not take account of ancestral polymorphism which should lead to older divergence time estimates than those here. These differences can be considerable [43], but whether or not this fully explains the observed differences in divergence time estimates will require further research.

## Conclusions

Analyses of the nuclear genome revealed evidence of ancient lineage divergence and secondary contact within the threatened *T. boettgeri* from Gran Canaria. Mixed ancestry was detected at areas that lineages meet, but this appears spatially restricted despite the short geographical distances. Limited admixture appears to facilitate identification of populations with single ancestry which we argue should be incorporated into conservation policy.

## Methods

### Sample sites

Fieldwork to capture individuals and sample tail tips was performed in Gran Canaria and El Hierro in May 2019, authorized by the Consejería de Política Territorial, Sostenibilidad y Seguridad, Gobierno de Canarias (permit 2018/30972 to the first author). This provided tail tips from 97 specimens from 29 sites on Gran Canaria and 7 specimens from 2 sites on El Hierro, preserved in DNA/RNA Shield™ solution (Zymo Research). *T. boettgeri* tail tips were also available from 18 specimens caught in June 2007 from 7 sites in Gran Canaria (with 1 of 3 sites being the same as a 2019 sample site), under permit 17,213 from the Consejería de Medio Ambiente y Aguas, Cabildo de Gran Canaria, to the first author and T. Tejangkura (all preserved in ethanol). Thirteen *T. boettgeri* were similarly sampled from the Selvagen Islands of Selvagem Grande and Selvagem Pequena in September 2017 under permit 09/IFCN/2017 issued by the Secretaria Regional do Ambiente e Recursos Naturais, Governo Regional da Madeira, to one of the authors (R. V.). Figure 1 provides sample site locations, and a full specimen list with latitudes and longitudes is provided in the Additional file: Table 1. All geckos used in the study were released at the site of capture. The Liverpool John Moores University ethics committee approved the research on 02/05/2019.

### Genotyping-by-sequencing

DNA extractions and genotyping-by-sequencing (GBS) were performed on all 135 specimens by Novogene using the protocol outlined here. Genomic libraries were prepared following DNA digestion with the restriction enzyme Mse I. Solexa P1 and P2 adapters (containing a 6-bp barcode sequence) were then added to the digested fragments, followed by a second digestion with the NlaIII restriction enzyme. Sequences with P1 and P2 adapters at both ends were PCR amplified; DNA fragments were pooled and size-selected using electrophoresis before purification. Illumina sequencing was used for paired-end 150-bp sequencing of the DNA fragments.

Adapters and barcodes were removed from the sequence reads. Paired reads were removed when fewer than 90% of bases could be read and/or when more than 50% of the single-end sequencing read contained low-quality bases.

### De novo whole genome sequencing

A whole *T. boettgeri* genome was sequenced de novo to provide a reference genome for alignment of the GBS reads. DNA was extracted from the tail tip of one additional *T. boettgeri* captured from site 50 (Additional file: Table 1 and Fig. 1). The tissue was frozen at −20 °C for 5 days and subsequently at − 80 °C until DNA was extracted using a Circulomics Nanobind Tissue Kit. The Centre for Genomic Research, University of Liverpool, then prepared a PacBio HiFi DNA library and performed whole genome sequencing on two Sequel II SMRT cells in CCS run mode.

The expected genome size was estimated using a *k-*mer analysis on the PacBio HiFi raw reads (here, *k* = 21) using jellyfish v.1.1.11 [44] and GenomeScope 2.0 [45]. The reads were assembled using hifiasm v.19.0 [46], and the completeness of the genic space was assessed using BUSCO v.5.2.2 [47] on a Sauropsida dataset that comprised 7480 BUSCO groups. A *k-*mer analysis was performed with merqury v.1.3 [48] to assess the quality of the assembled sequence by comparing the *k*-mers present in the reads with the *k*-mers present in the assembled genome. Telomere identification was performed by checking the ends (1 kbps) of each contig for an enriched presence of the telomeric sequence 5′-TTAGGG-3′. De novo repetitive sequence annotation was performed using EDTA v.2.0 [49], and RepeatMasker v.4.1.2 [50] was used to soft mask the whole genome.

Coding gene prediction was performed separately for each haplotype using Augustus v3.4 [51] and the MAKER v.3.01 pipeline [52] (comprising GeneMark-ES, Augustus, and EVidenceModeler), trained with Gekkota proteins downloaded from NCBI RefSeq. The two predictions were merged according to the results of the GeneValidator v.2.1.12 tool [53] which retained only the best predictions for every gene site.

### Alignment of sequencing reads and SNP calling

Reads were aligned to the reference genome using *ipyrad* (ver. 0.9.93) [54]. One individual from Selvagem Grande was removed due to a low number of aligned reads, leaving 134 individuals for analyses. Default values were specified in the *ipyrad* parameters file with the following exceptions: the maximum cluster depth within samples was set to 300, the minimum length of reads after adapter trim was set to 130, the maximum number of unknown bases in the consensus was 0.01, the minimum number of samples per locus for the locus to be Output was 101 (75%), the maximum number (proportion) of SNPs per locus was 0.25, the maximum number of indels per locus was 5, and the maximum heterozygous sites per locus was 0.25. Three bases were trimmed from the start of both R1 and R2 reads.

Following alignment, genomic data were output in two formats: (i) the entire sequences obtained from each sequenced tag (the tags were then subsampled to reduce computational requirements for analyses: see below) and (ii) the full set of SNPs called from the sequence tags, which was also subsampled to obtain a thinned set of SNPs, consisting of one SNP per tag, using the script subsetSNPs.py (https://github.com/ksil91/Scripts/blob/master/subsetSNPs.py). Prior to subsampling, the full set of SNPs was filtered using VCFtools v. 0.1.15 [55] with the following settings: minor allele count ≥ 1, maximum and minimum number of alleles = 2, exclusion of sites with < 85% of data, and exclusion of sites with mean depth values (over all included individuals) ≤ 5 or ≥ 100.

### Analyses of admixture

Detection of genomic clusters and degrees of admixture (ancestry coefficients) were estimated using the R package LEA v.2.0 [56]. Here and elsewhere, R version 4.2 was used [57]. The number of ancestral population clusters within the thinned SNPs dataset was assessed using the cross-entropy criterion in the snmf function, for *K* = 1–15 ancestral populations (10 replicates for each K). The matrix of ancestry coefficients was then calculated for the selected value of K.

The Bayesian approach recently implemented in the program BPP v.4.7 in A00 mode (MSci model C in [58]) was used to further analyse admixture and estimate uncertainty associated with introgression probabilities using DNA sequences as opposed to the SNPs [59, 60]. BPP v.4.7 contains its own phasing algorithm and also, for analyses of introgression, favours use of more DNA sequence rather than sampling more individuals (with one individual being sufficient), making it very suitable here [60]. Nonetheless, analyses of admixture within a specified evolutionary history can be extremely computationally intensive which precluded global analyses across all specimens. Hence, samples of *n* ≥ 3 single ancestry individuals (defined by the LEA analysis above as having an ancestry coefficient ≥ 95% for one cluster) were selected from a single sample site. Similar single ancestry individuals (*n* ≥ 3) were then selected from a site in a neighbouring area corresponding to a different genomic cluster. Individuals (*n* ≥ 3) from a third intermediate site (or two adjacent sites in one case) located between the two single ancestry sites were also selected (Additional file: Fig. S1). Individuals from pairs of genomic clusters with adjacent geographical distributions were all tested in this way. In the presence of bidirectional introgression and equal geographical distances between the single ancestry and intermediate groups, introgression probabilities of 50% into the intermediate site would be expected, on average. To reduce computation time, 2000 sequencing tag alignments were subsampled from the full set of tag alignments.

**Fig. 7:**
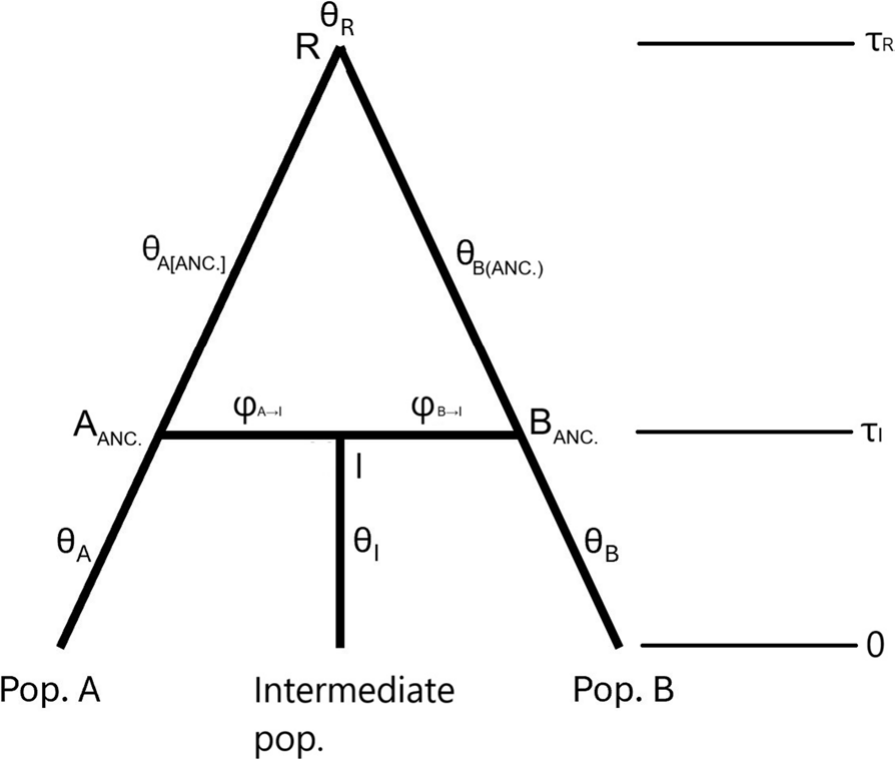
Graphical summary of the model used for Bayesian analysis of introgression. Lineages A and B diverge at time *τ*_R_ and evolve into populations A and B. The two lineages come into contact to form the hybrid, I, at time *τ*_I_. Introgression probabilities into I at time *τ*_I_ are given as *φ*_A→I_ and *φ*_B→I_. The hybrid, I, evolves into the intermediate population sampled in our analysis. *θ*s are used to denote population size parameters throughout

The tree was fixed as a bifurcating topology in the BPP introgression analysis, with two tips representing the two single ancestry sample sites and an intermediate third tip created by their hybridization (see Fig. 7). This modelled the secondary contact of the two lineages within each pair and their subsequent admixture to form a hybrid population. In this and other BPP analyses (see later) as follows: (i) population size parameters (θs) were integrated out to improve convergence, and (ii) the Jukes-Cantor model of DNA substitution was specified because it provided the best fit in most cases when tested on a subsample of 30 loci. Following preliminary runs, the prior on the divergence time of the root (τ_R_) was specified from the inverse gamma distribution: InvG (4, 0.01). This is a diffuse prior with a variance of 5.5 × 10^−6^ and a mean of 0.003, which equates to 0.3% sequence divergence (approximating average sequence divergence between the root and tips of the species tree). The prior on the introgression probability was specified from the beta distribution: *β*(1, 1). This is suitable when there is no prior information about introgression because it provides a constant probability density function across the interval [0, 1]. The first 25,000 steps of the MCMC chains were disregarded as burn-in, while the remaining 1.5 million steps were sampled at 10 iteration intervals and log-likelihoods examined to ensure convergence. Two or three replicate analyses, starting from different values, were also used to check convergence on the same posterior.

### Phylogenomic inference

Estimation of the phylogenomic history was achieved using just selected individuals (*n* = 3 for each genomic cluster) that corresponded to individuals with single ancestry coefficients ≥ 95% in the LEA analysis. All genomic clusters detected by LEA were included. The aim was to minimize the impact of gene flow on the analysis. The evolutionary histories of these individuals/groups were then inferred using several methods, described below.

First, all SNPs were concatenated for each individual, and the relationships among them were inferred using the maximum likelihood (ML) approach implemented in RAXML-NG v.1.2.1 [61]. The GTGTR4 unphased diploid genotypes model with Lewis ascertainment bias corrections was specified. Analyses were performed on 1000 bootstrap replicates of the data, and a consensus tree was calculated using the same program.

Second, a species tree was inferred from all SNPs using SVDquartets [62] within PAUP (ver. 4.0a) [63]. As elsewhere, “species” were defined from genomic clusters identified by the LEA analysis of ancestry coefficients. The QFM tree assembly algorithm was used, with all possible quartets evaluated. Support for branches was assessed from 1000 bootstrap replicates.

Finally, BPP v.4.7 was used to infer species trees from 2000 subsampled sequenced tags (i.e. variable and invariant sites, with each tag specified as a locus), using the BPP A01 analysis [59]. Population size parameters were integrated out. Following preliminary runs, the prior on the divergence time of the root was specified from the same inverse gamma distribution already described for the BPP analyses of introgression, i.e. InvG (*α* = 4, *β* = 0.01). MCMC chains were run for 1.5 million steps, sampled every 10 steps (with a burn-in period of 50,000 steps). Analyses were replicated three times, each from a different starting point.

Bayes factors were calculated from the respective marginal likelihoods of species histories with posterior probabilities (PP) ≥ 0.05 in the BPP A01 analysis [64]. This was achieved using BPP v.4.7 to obtain power posteriors for each species history. A power posterior is the posterior with the likelihood raised to a power *b* and equivalent to the posterior distribution when *b* = 1.0 and to the prior distribution when *b* = 0.0. A sample of 1000 sequenced tags were used for these analyses to reduce computational requirements. For each species history, eight MCMC chains were computed, with the likelihood raised to a different value of *b* in each case. The R package bppr v. 0.6.3 [65] was used to determine suitable values of *b* and also to calculate marginal likelihoods and Bayes factors.

### Divergence time estimation

The favoured species tree topology from the BPP A01 analysis was used to estimate divergence times from 2000 subsampled loci, using the BPP A00 model [59]. Posterior MCMC samples of divergence times were obtained from BPP and transformed to geological times using a calibration on the divergence time of the El Hierro populations from its sister lineage (R package bppr v.0.6.3 [43, 65]). This calibration was specified from the uniform prior, U (0.7, 1.2). The rationale for this prior was as follows: (i) dating of subaerial rocks has demonstrated that emergence of El Hierro occurred about 1.2 Ma [66], and (ii) mtDNA divergence between the El Hierro population and the most closely related extant *T. boettgeri* is considerable [23, 24, 39], and formal mtDNA divergence time dating (using an approximate mtDNA rate) suggests that El Hierro was colonized soon after it appeared [24]. Our calibration assumed direct colonization of El Hierro from an ancestor shared with an extant lineage 0–0.5 Ma after subaerial emergence of El Hierro.

## Supplementary Information

Additional File: Table 1 and Figures S1-S3. Table 1 - Locations of sample sites and years of capture. Fig. S1 - Map of sites analysed in BPP introgression analyses. Fig. S2 - De novo genome sequencing of T.boettgerii Fig. S3 - Mean cross-entropies for different numbers of genomic clusters in LEA analysis

## Data Availability

The genomic data in this manuscript are available under NCBI bioproject PRJNA1281674. https://www.ncbi.nlm.nih.gov/sra/?term= PRJNA1281674. The NCBI Biosample accessions for the individuals used in the GBS analysis are SAMN49559779 to SAMN49559912. The haplotypes of the genome assembly are provided in NCBI bioprojects PRJNA1279588 and PRJNA1279589.
