# Supplementary material for: Genomic identification of conservation areas amid lineage divergence and admixture in a threatened island gecko
Source: BMC Biol. 2025 Oct 22;23:317. doi: 10.1186/s12915-025-02394-6 (PMC12542033; doi:10.1186/s12915-025-02394-6)

**Supplemental Information for:**

**Genomic identification of conservation areas amid** **lineage divergence and admixture in a threatened island gecko**

**Brown, R.P. , Bianco, L., Fontana, P. , Shum, P. , Vasconcelos, R., Jin, Y.**

**Table of Contents:**

| Table 1 | Page 2 |
| --- | --- |
| Figure S1 | Page 3 |
| Figure S2 | Page 4 |
| Figure S3 | Page 5 |

**Supplementary Table 1.** Locations of sample sites given in decimal degrees. Sites represented solely by numbers are within Gran Canaria. Coordinates for the Selvagens correspond to general coordinates for Selvagem Grande (SG) and Selvagem Pequena (SP), and not the exact site of capture within these islands.

| **Sample site** | **Latitude** | **Longitude** | **Number of specimens** | **Year of capture** |
| --- | --- | --- | --- | --- |
| 5 | 27.8859 | -15.4398 | 2 | 2007 |
| 6 | 27.8537 | -15.4683 | 4 | 2007 |
| 7 | 27.8111 | -15.4819 | 2 | 2007 |
| 12 | 27.7798 | -15.6231 | 5 | 2007 |
| 17 | 28.1497 | -15.6944 | 1 | 2007 |
| 20 | 28.1330 | -15.4865 | 5 | 2019 |
| 21 | 28.1492 | -15.5400 | 5 | 2019 |
| 22 | 28.1438 | -15.5978 | 3 | 2019 |
| 23 | 28.1400 | -15.6372 | 5 | 2019 |
| 24 | 28.1058 | -15.7025 | 5 | 2019 |
| 25 | 28.0454 | -15.7297 | 5 | 2019 |
| 26 | 27.9974 | -15.8141 | 5 | 2019 |
| 27 | 27.9257 | -15.7580 | 5 | 2019 |
| 28 | 27.8700 | -15.7347 | 2 | 2019 |
| 29 | 27.8413 | -15.7469 | 5 | 2019 |
| 30 | 27.8499 | -15.5659 | 4 | 2019 |
| 31 | 27.9096 | -15.5396 | 4 | 2019 |
| 32 | 27.9084 | -15.6340 | 3 | 2019 |
| 33 | 28.1647 | -15.4346 | 5 | 2019 |
| 34 | 28.0743 | -15.4299 | 5 | 2019 |
| 35 | 27.9976 | -15.4084 | 4 | 2019 |
| 36 | 28.0902 | -15.4949 | 3 | 2019 |
| 37 | 28.1155 | -15.5864 | 1 | 2019 |
| 38 | 28.0681 | -15.6586 | 1 | 2019 |
| 39 | 27.9939 | -15.4808 | 1 | 2019 |
| 40 | 27.9310 | -15.6570 | 4 | 2019 |
| 41 | 27.9495 | -15.3919 | 3 | 2019 |
| 42 | 27.9263 | -15.4310 | 2 | 2019 |
| 43 | 27.8619 | -15.3906 | 3 | 2019 |
| 44 | 27.8116 | -15.4672 | 2 | 2019 |
| 45 | 27.8031 | -15.4786 | 2 | 2019 |
| 46 | 27.7657 | -15.5699 | 3 | 2007&2019 |
| 47 | 27.7587 | -15.6728 | 2 | 2019 |
| 48 | 27.9928 | -15.7084 | 2 | 2019 |
| 49 | 28.0455 | -15.4208 | 1 | 2007 |
| 50 | 27.9494 | -15.3842 | 1 | 2007 |
| Hierro 1 | 27.7510 | -18.0192 | 5 | 2019 |
| Hierro 2 | 27.8370 | -17.9474 | 2 | 2019 |
| Selvagem Grande (SG) | 30.1446 | -15.8632 | 5 | 2017 |
| Selvagem Pequena (SP) | 30.0336 | -16.0292 | 7 | 2017 |

**Figure S1.** Map of sites analysed in BPP introgression analyses. Sites that were analyzed in each analysis are connected by lines, coloured according to main genomic clusters. Groups of sites used in each analysis are found within the black rectangles. For example, sites 23, 25, 32 were analyzed in one analysis with 25 being the intermediate site.


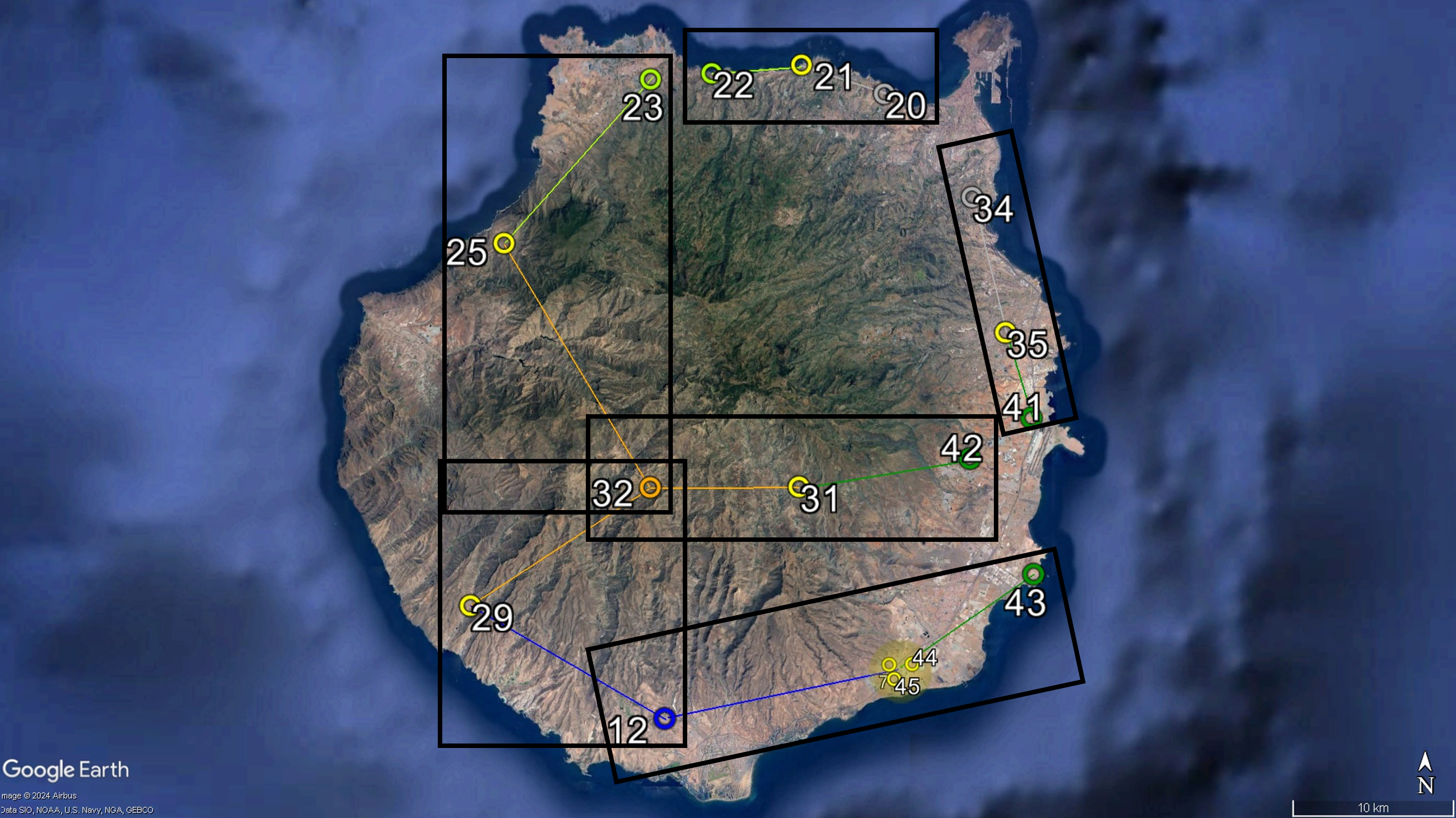


**Figure S2: De novo genome sequencing of *T.boettgerii***

**A. Kmer analysis for genome size estimation**


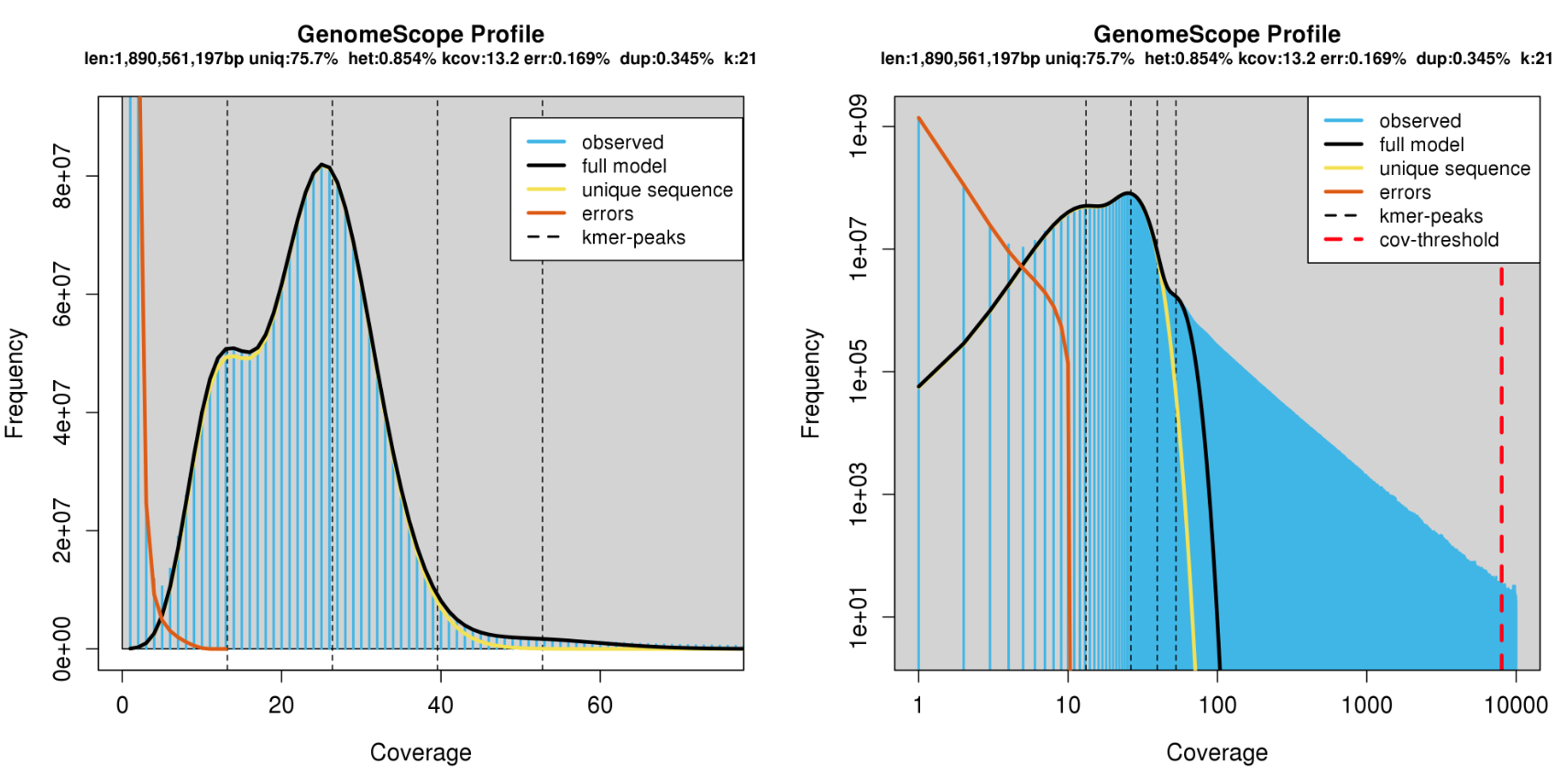


**B. Merqury analysis of reads vs assembled sequence**


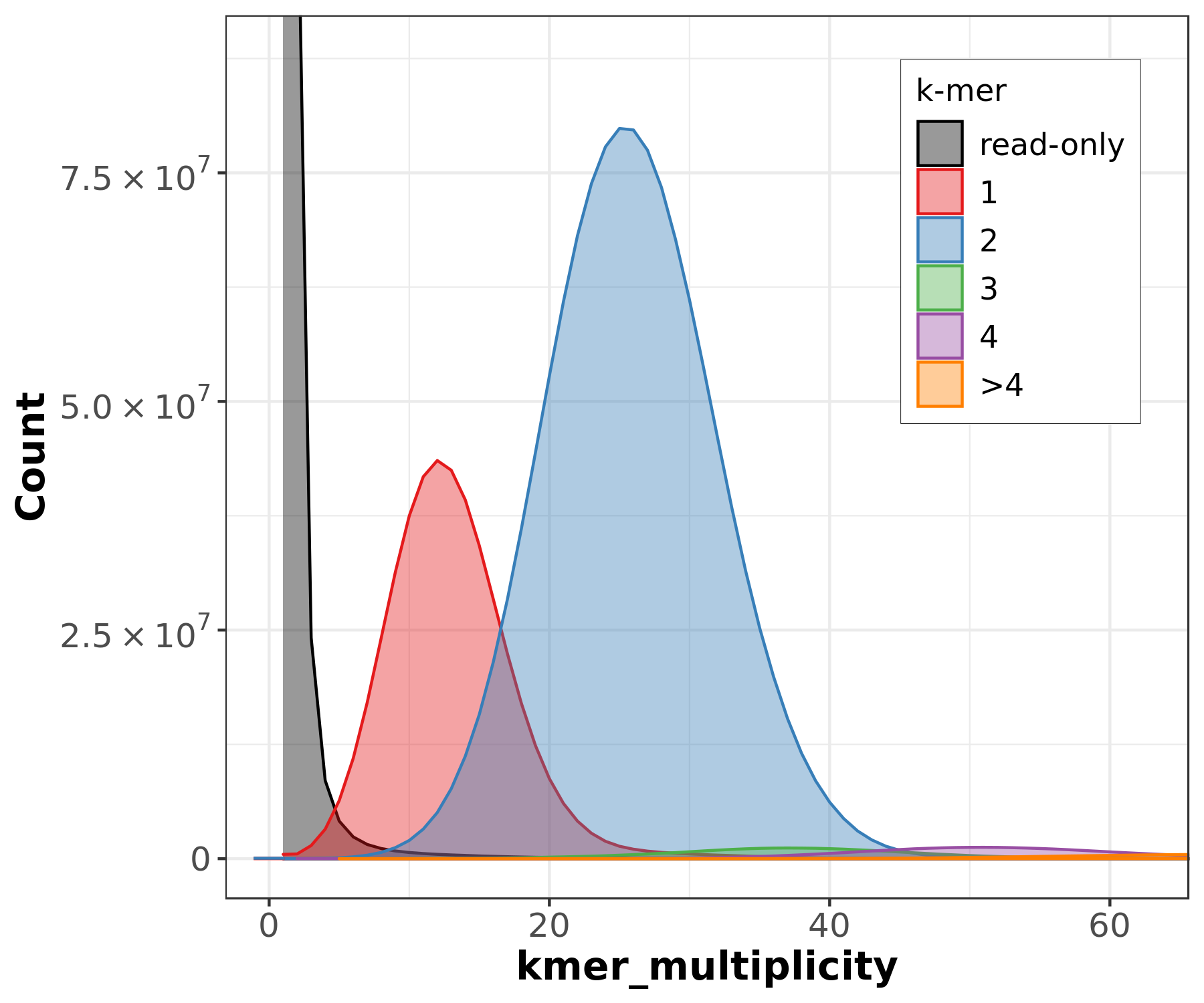

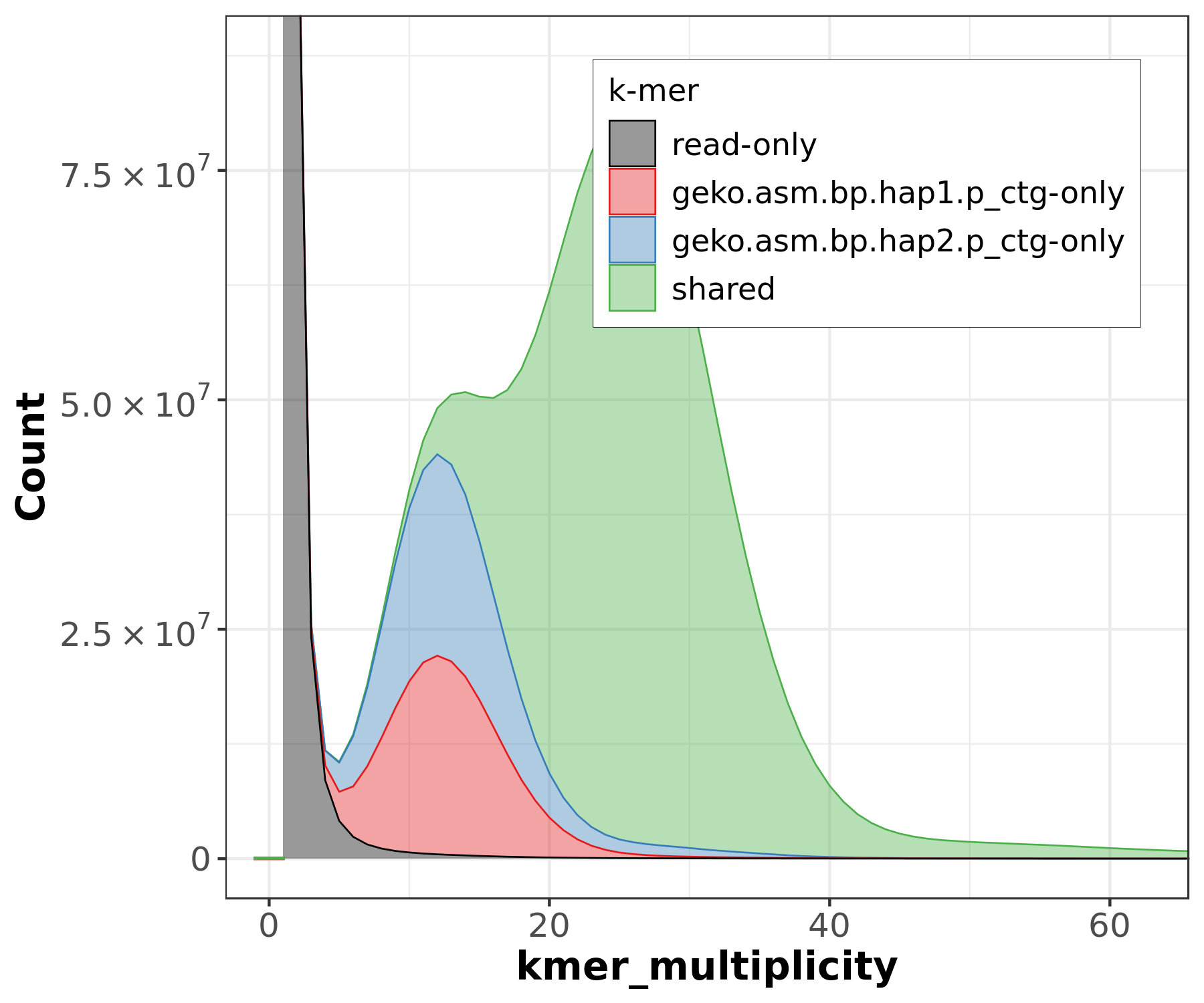


Genome Assembly kmers tot. kmers %

geko.asm.bp.hap1.p_ctg all 1517940603 1759406517 86.2757%

geko.asm.bp.hap2.p_ctg all 1533603947 1759406517 87.166%

both all 1748495929 1759406517 99.3799%

**Figure S3.** Plot from LEA analysis genomic structure showing mean cross-entropies for different numbers of ancestral populations in the model. The lowest mean cross-entropies are observed when there are 5,6 or 7 ancestral populations in the model.


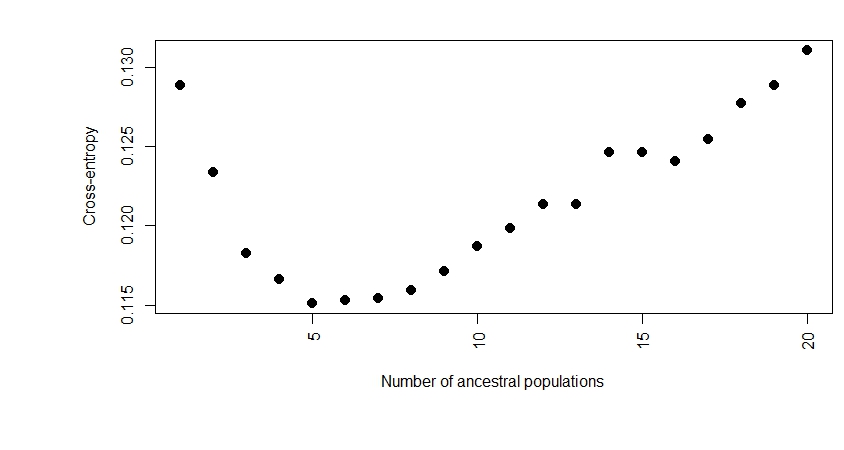

Supplement: Supplementary file 1 — Additional File: Table 1 and Figures S1-S3. Table 1 - Locations of sample sites and years of capture. Fig. S1 - Map of sites analysed in BPP introgression analyses. Fig. S2 - De novo genome sequencing of T.boettgerii Fig. S3 - Mean cross-entropies for different numbers of genomic clusters in LEA analysis [file 12915_2025_2394_MOESM1_ESM.docx]
